# Rabring7 Degrades c-Myc through Complex Formation with MM-1

**DOI:** 10.1371/journal.pone.0041891

**Published:** 2012-07-23

**Authors:** Rina Narita, Hirotake Kitaura, Ayako Torii, Erika Tashiro, Makoto Miyazawa, Hiroyoshi Ariga, Sanae M. M. Iguchi-Ariga

**Affiliations:** 1 Graduate School of Agriculture, Hokkaido University, Sapporo, Japan; 2 Graduate School of Pharmaceutical Sciences, Hokkaido University, Sapporo, Japan; Wayne State University, United States of America

## Abstract

We have reported that a novel c-Myc-binding protein, MM-1, repressed E-box-dependent transcription and transforming activities of c-Myc and that a mutation of A157R in MM-1, which is often observed in patients with leukemia or lymphoma, abrogated all of the repressive activities of MM-1 toward c-Myc, indicating that MM-1 is a novel tumor suppressor. MM-1 also binds to the ubiquitin-proteasome system, leading to degradation of c-Myc. In this study, we identified Rabring7, a Rab7-binding and RING finger-containing protein, as an MM-1-binding protein, and we found that Rabring7 mono-ubiquitinated MM-1 in the cytoplasm without degradation of MM-1. Rabring7 was also found to bind to c-Myc and to ubiquitinate c-Myc in a threonine 58-dependent manner. When c-Myc was co-transfected with MM-1 and Rabring7, c-Myc was degraded. Furthermore, it was found that c-Myc was stabilized in MM-1-knockdown cells even when Rabring7 was transfected and that Rabring7 was bound to and co-localized with MM-1 and c-Myc after MM-1 and Rabring7 had been translocated from the cytoplasm to the nucleus. These results suggest that Rabring7 stimulates c-Myc degradation via mono-ubiquitination of MM-1.

## Introduction

The proto-oncogene product c-Myc plays pivotal roles in cell proliferation, differentiation and apoptosis induction, and its expression level is tightly regulated at several steps, including transcription, translation and protein stability [1]–[4]. The half life of c-Myc is about 30 min [1], [5], and c-Myc is degraded by the ubiquitin-protease system [6], [7]. It has been reported that Skp2, an F-box protein, binds both to the myc box II and to b-HLH domains located in the N-proximal and C-terminal regions, respectively, of c-Myc to facilitate ubiquitination of c-Myc [8], [9]. Furthermore, it has been reported that Fbw7, another F-box protein, facilitates degradation of c-Myc through phosphorylation of serine at amino acid number 62, which is present in the myc box I, followed by phosphorylation of threonine at amino acid number 58 (T58) [10]. These findings indicate that c-Myc is degraded through various pathways.

Functions of c-Myc are modulated by its interacting proteins, giving rise to the versatile functions of c-Myc. We have reported that MM-1, a novel protein binding to the myc box II, suppressed transcription and transformation activities of c-Myc and that A157R mutation of c-Myc, which is observed at high frequency in patients with lymphoma or leukemia, abrogated all of the functions of MM-1 toward c-Myc, indicating that MM-1 is a novel tumor suppressor [11], [12]. As to the MM-1-dependent transrepression pathway of c-Myc, we have shown that MM-1 recruited the HDAC complex to c-Myc via TIF1β, a corepressor [13], and that the c-*fms* gene is a target gene for this pathway [14], [15]. MM-1 has been shown to bind to p73α to enhance growth-repression activity of p73 [16].

The *mm*-1 gene is located at the q12–q13 region of chromosome 12 and produces 4 splicing isoforms, α, β, γ and δ [17]. Of the 4 subunits, MM-1α has been shown to be a subunit of prefoldin, prefoldin 5/Gim5 [18], [19] Prefoldin is composed of 6 subunits and plays a role in bringing newly synthesized proteins such as actin or tubulin to Tric/CCT, in which proteins are correctly folded [20] suggesting that MM-1 participates in protein folding. It has been reported that prefoldin3/VBP1 facilitates the ubiquitin/proteasome-dependent degradation of HIV integrase by binding to the ubiquitin E3 ligase complex composed of Cullin2 and von Hippel-Lindau (VHL), suggesting contribution of prefoldin to the function of the ubiquitin/proteasome system [21]. We have shown that MM-1 binds to Rpt3 and Rpn12, subunits of the proteasome, to ubiquitinate c-Myc, resulting in degradation of c-Myc [22], and we have also shown that the expression of prefoldin subunits is mutually regulated and that the free form of prefoldin subunits tends to be degraded through the ubiquitin/proteasome system [23]. These findings, including ours, suggest a role of MM-1 in protein degradation.

In this study, we identified Rabring7 as an MM-1-binding protein. Raring7, also named BCA2, is a protein containing a RING finger domain, which is a characteristic feature of ubiquitin E3 ligase [24], [25]. Our results showed that Rabring7 facilitated degradation of c-Myc via complex formation with MM-1.

## Results

### Identification of Rabring7 as an MM-1-binding protein

**Figure 1:**
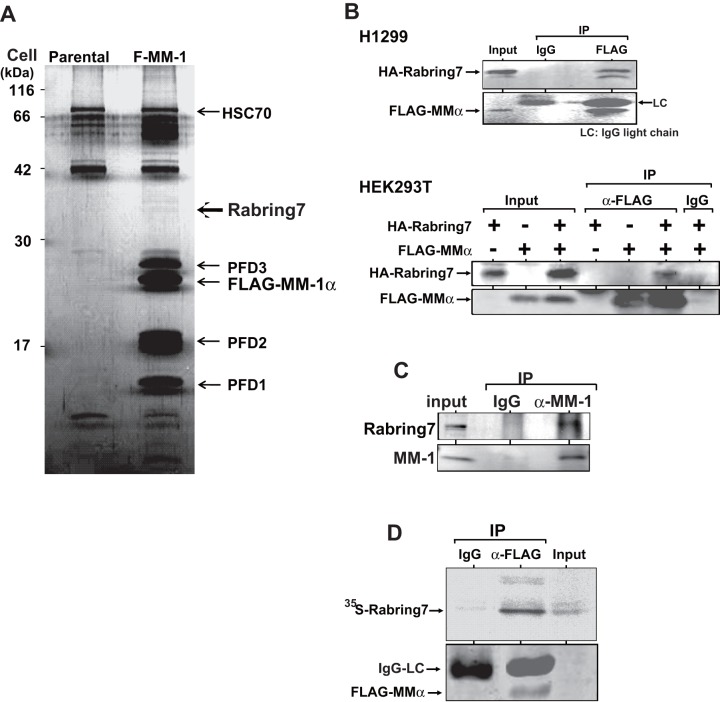
Identification of Rabring7 as an MM-1-binding protein. **A**. Proteins prepared from H1299 cells and F-MM-1-expressing H1299 cells were immunoprecipitated with an anti-FLAG antibody, and the precipitates were subjected to TOF-MS analyses as described in Experimental procedures. **B**. H1299 cells (upper panel) and HEK293 cells (lower panel) were transfected with various combinations of expression vectors for HA-Rabring7 and FLAG-MM-1α. At 48 hrs after transfection, proteins prepared from cells were immunoprecipitated with an anti-FLAG antibody or with IgG, and the precipitates were analyzed by Western blotting with anti-HA and anti-FLAG antibodies. **C**. Proteins prepared from HEK293T cells were immunoprecipitated with an anti-MM-1 antibody or with IgG, and the precipitates were analyzed by Western blotting with anti-Rabring7 and anti-MM-1 antibodies. **D**. FLAG-MM-1 expressed in and purified from *E. coli* was reacted with ^35^S-labeled Rabring7 that had been synthesized by using reticulocyte lysates. Proteins in a reaction mixture were then immunoprecipitated with an anti-FLAG antibody or with IgG, and the precipitates were separated on a gel followed by fluorography.

To identify MM-1-binding proteins, H1299 cells expressing FLAG-tagged MM-1α were established. Proteins were extracted from H1299 cells expressing FLAG-tagged MM-1α and parental H1299 cells and immunoprecipitated with an anti-FLAG antibody, and the precipitates were then separated in a polyacrylamide gel and silver-stained. Proteins observed in FLAG-MM-1α-expressing cells but not in parental H1299 cells were cut out and subjected to TOF-MS analyses (Figure 1A). In addition to three subunits of prefoldin, PFD1, PFD2 and PFD3, a faint but specific protein band was found to be Rabring7. Rabring7, also named BCA2, was identified as a Rab7-binding protein [24] and is over-expressed in breast cancer cells [25], [26]. Rabring7 contains Zinc finger and RING finger domains in the N-terminus and C-terminus, respectively, and is ubiquitinated by itself in the Zinc finger domain, indicating that Rabring7 is a ubiquitin ligase [25]–[27]. To confirm the association of MM-1α with Rabring7, human H1299 and HEK293T cells were transfected with expression vectors for FLAG-MM-1α and HA-Rabring7. Forty-eight hrs after transfection, proteins prepared from transfected cells were immunoprecipitated with an anti-FLAG antibody or with non-specific IgG, and the precipitates were analyzed by Western blotting with anti-HA and anti-FLAG antibodies. As shown in Figure 1B, HA-Rabring7 was immunoprecipitated with the anti-FLAG antibody but not with IgG, indicating the association of FLAG-MM-1α with HA-Rabring7. Interaction of FLAG-MM-1α with HA-Rabring7 was also confirmed in HEK293 cells into which HA-Rabring7 had been co-transfected with various combinations of FLAG-MM-1α (Figure 1B, HEK293
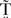
To examine endogenous association of MM-1α with Rabring7, proteins extracted from HEK293 cells were immunoprecipitated with an anti-MM-1 antibody or with normal goat IgG and subjected to Western blotting with anti-MM-1 and anti-Rabring7 antibodies. As shown in Figure 1C, Rabring7 was immunoprecipitated with the anti-MM-1 antibody, indicating endogenous association of MM-1α with Rabring7. To determine whether the interaction of MM-1α with Rabring7 is direct or indirect, purified FLAG-MM-1α was reacted with ^35^S-labeled Rabring7 that had been synthesized *in vitro* using a coupled transcription-translation system. Proteins in the mixture were then immunoprecipitated with the anti-FLAG antibody or with IgG, and the precipitates were separated on a gel and visualized by fluorography (Figure 1D). The results clearly showed that ^35^S-Rabring7 was specifically immunoprecipitated with the anti-FLAG antibody, indicating direct interaction of MM-1α with Rabring7.

### Mono-ubiquitination of MM-1 α by Rabring7

**Figure 2:**
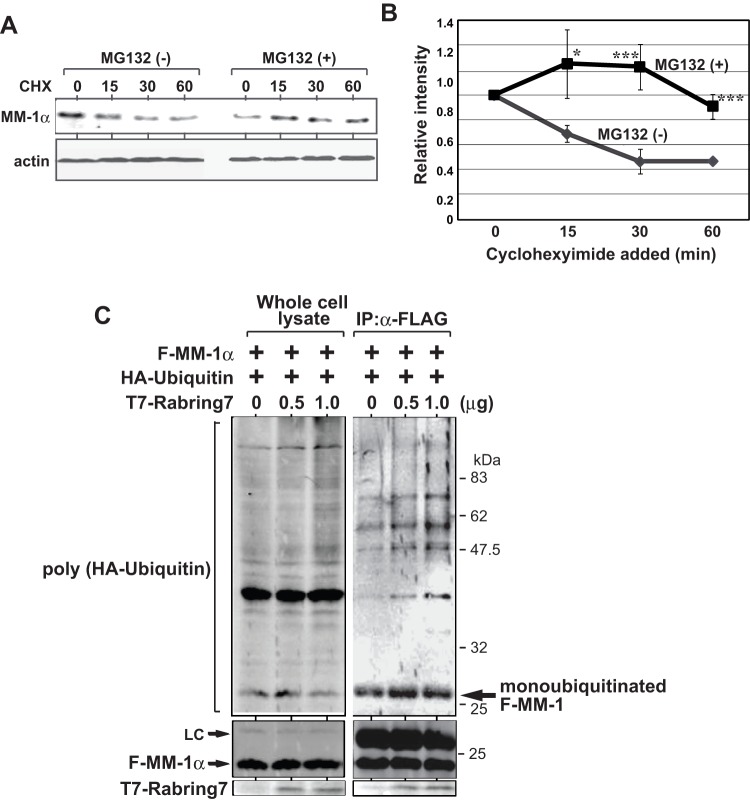
Mono-ubiquitination of MM-1 by Rabring7. **A**. HeLa cells were cultured in the presence or absence of cycloheximide. At the times indicated, the expression levels of MM-1α and actin in cells were examined by Western blotting with respective antibodies. **B**. The intensity of bands corresponding to MM-1α and to actin in Fig. 2A was quantified, and relative intensity of MM-1α to that of actin is shown. Values are means ± S.D. n = 3 experiments. Significance: *p<0.05 and ***p<0.001. **C**. HEK293T cells were transfected with FLAG-MM-1α together with HA-ubiquitin and with various concentrations of T7-Rabring7. Forty-eight hrs after transfection, proteins prepared from transfected cells were immunoprecipitated with an anti-FLAG antibody, and the precipitates were analyzed by Western blotting with anti-HA, anti-FLAG and anti-T7 antibodies (right panel). A part of the proteins prepared from transfected cells was also analyzed (left panel).

We first examined the half life of MM-1α. To do this, HeLa cells were cultured in the presence of cycloheximide, an inhibitor for protein synthesis, with or without MG132, an inhibitor for the proteasome, and MM-1α was analyzed by Western blotting with an anti-MM-1 antibody. As shown in Fig. 2A and its quantified results (Figure 2B), MG132 stabilized MM-1α, but MM-1α was not completely degraded in the absence of MG132, suggesting that MM-1α is partly degraded by the ubiquitin/proteasome system and that MM-1α is a relatively stable protein as described previously [23].

To examine whether MM-1α is ubiquitinated, HEK293T cells were transfected with FLAG-MM-1α together with HA-ubiquitin and with various concentrations of T7-Rabring7. Forty-eight hrs after transfection, proteins prepared from transfected cells were immunoprecipitated with an anti-FLAG antibody, and the precipitates were analyzed by Western blotting with an anti-HA antibody. Ladder bands corresponding to poly-ubiquitinated proteins were observed and the intensity of the bands was slightly increased with increasing doses of T7-Rabring7, indicating that Rabring7 worked as an E3 ligase toward MM-1α (Figure 2C). Furthermore, a clear band at more than 25 kDa, which may be the band corresponding to mono-ubiquitinated MM-1α, was observed, and the level of this band was also increased by Rabring7 (Figure 2C), suggesting that mono-ubiquitination of MM-1α occurred more strongly than poly-ubiquitination by Rabring7.

**Figure 3:**
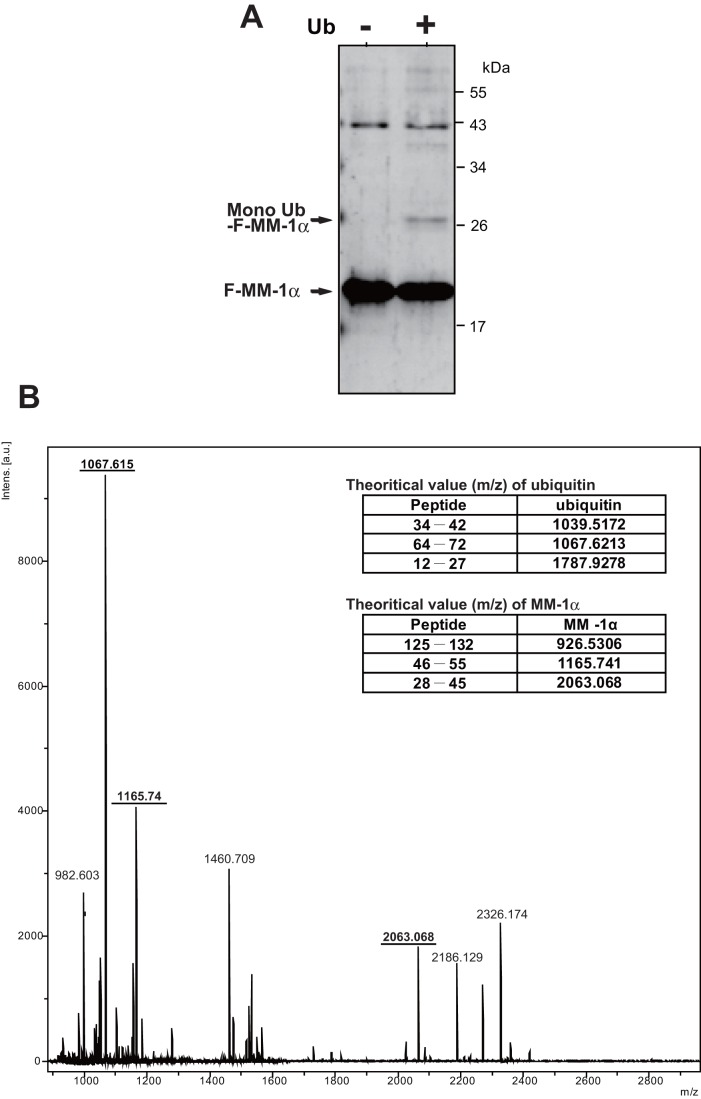
Identification of mono-ubiquitination of MM-1 by TOF-MS. **A**. Ubiquitination assays were carried out *in vitro* using E1, E2 and E3 mixtures and FLAG-MM-1 as a template as described in Experimental procedures. Proteins in the mixture were analyzed by Western blotting with an anti-FLAG antibody. **B**. After ubiquitination assays as described in the legend of Fig. 3A, proteins in the mixture were stained with coomassie brilliant blue. Stained mono-ubiquitinated FLAG-MM-1 was cut out and then subjected to TOF-MS analysis as described in Experimental procedures.

Ubiquitination of MM-1α was further confirmed by using an *in vitro* ubiquitination assay. Recombinant FLAG-MM-1α purified from *E. coli* was reacted with enzyme mixtures containing all of the proteins necessary for ubiquitination such as E1, E2 and E3 enzymes in the presence or absence of ubiquitin. Proteins in the mixture were analyzed by Western blotting with an anti-FLAG antibody. As shown in Figure 3A, in addition to faint bands corresponding to poly-ubiquitinated FLAG-MM-1α, a clear band, the molecular mass of which may be mono-ubiquitinated FLAG-MM-1α, was observed, and it was cut out and subjected to TOF-MS analyses. FLAG-MM-1α without ubiquitination reaction was also analyzed by TOF-MS as a control. As shown in Figure 3B, a mono-ubiquitinated peptide with a molecular mass of 1067.615 was observed, confirming mono-ubiquitination of MM-1α.

### Association of c-Myc with Rabring7

**Figure 4:**
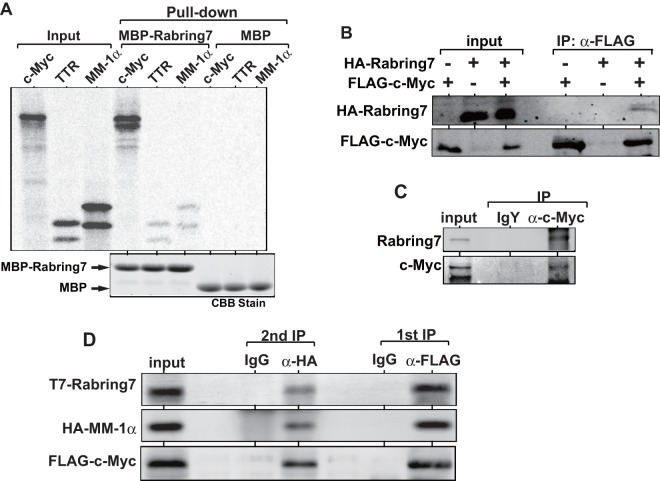
Binding of Rabring7 to c-Myc. **A**. MBP-Rabring7 or MBP expressed in and purified from *E. coli* was reacted with ^35^S-labeled c-Myc, transthyretin (TTR) and MM-1α, and pull-down assays were carried out as described in Experimental procedures. **B**. HEK293 cells were transfected with various combinations of expression vectors for HA-Rabring7 and FLAG-c-Myc. At 48 hrs after transfection, proteins prepared from cells were immunoprecipitated with an anti-FLAG antibody, and the precipitates were analyzed by Western blotting with anti-HA and anti-FLAG antibodies. **C**. Proteins prepared from HEK293T cells were immunoprecipitated with an anti-c-Myc antibody or with IgY, and the precipitates were analyzed by Western blotting with anti-Rabring7 and anti-c-Myc antibodies. **D**. H1299 cells were co-transfected with expression vectors for T7-Rabring7, HA-MM-1α and FLAG-c-Myc. At 48 hrs after transfection, proteins extracted from cells were immunoprecipitated with an agarose-conjugated anti-FLAG antibody and the proteins bound to agarose bead were eluted with FLAG peptide. The eluted proteins were then immunoprecipitated with an anti-HA antibody and precipitates were analyzed by Western blotting with anti-T7, anti-HA and anti-FLAG antibodies as described in Experimental procedures.

Since MM-1α is a c-Myc-binding protein, we then examined whether Rabring7 associates with c-Myc in addition to MM-1α. To do this, *in vitro* pull-down assays were carried out using recombinant MBP or MBP-Rabring7 and ^35^S-labeled c-Myc that had been synthesized *in vitro*. ^35^S-labeled MM-1α was used as a positive control. Rabring7 was identified as a Rab7-binding protein. Rab7 facilitates trafficking of proteins through the endosome to lysosome or to the membrane. Rabring7 is localized in the endosome. Since transthyretin (TTR) is synthesized in the cytoplasm and secreted, we used ^35^S-labeled TTR as positive control that would bind to Rabring7. As shown in Figure 4A, c-Myc directly bound to MBP-Rabring7 and the degree of binding activity of c-Myc to MBP-Rabring7 was higher than that of MM-1α. c-Myc did not bind to MBP itself (Figure 4A) as shown previously [28]. Association of Rabring7 and c-Myc in cells was then examined. First, HEK293 cells were transfected with HA-Rabring7 and with FLAG-c-Myc. Forty-eight hrs after transfection, proteins prepared from cells were immunoprecipitated with an anti-FLAG antibody, and the precipitates were analyzed by Western blotting with anti-HA and anti-FLAG antibodies. The results showed that the anti-FLAG antibody immunoprecipitated HA-Rabring7 (Figure 4B). To examine endogenous association of c-Myc with Rabring7, proteins from HEK293 cells were immunoprecipitated with a chicken anti-c-Myc antibody or with non-specific IgY, and the precipitates were analyzed by Western blotting with anti-Rabring7 and anti-c-Myc antibodies. As shown in Figure 4C, Rabrin7 was immunoprecipitated with the anti-c-Myc antibody but not with IgY, indicating that Rabrin7 was associated with c-Myc in cells. To examine a ternary complex of c-Myc with MM-1-1α and Rabring7, H1299 cells were transfected with FLAG-c-Myc together with T7-Rabring7 and HA-MM-1. At 48 hrs after transfection, proteins prepared from transfected cells were first immunoprecipitated with an agarose-conjugated anti-FLAG antibody, and the precipitates were then eluted from agarose by FLAG peptide. Proteins in elutes were again immunoprecipitated with an anti-HA antibody, and the precipitates were analyzed by Western blotting with anti-T7, anti-HA and anti-FLAG antibodies. The results showed that all three proteins were co-immunoprecipitated (Figure 4D).

**Figure 5:**
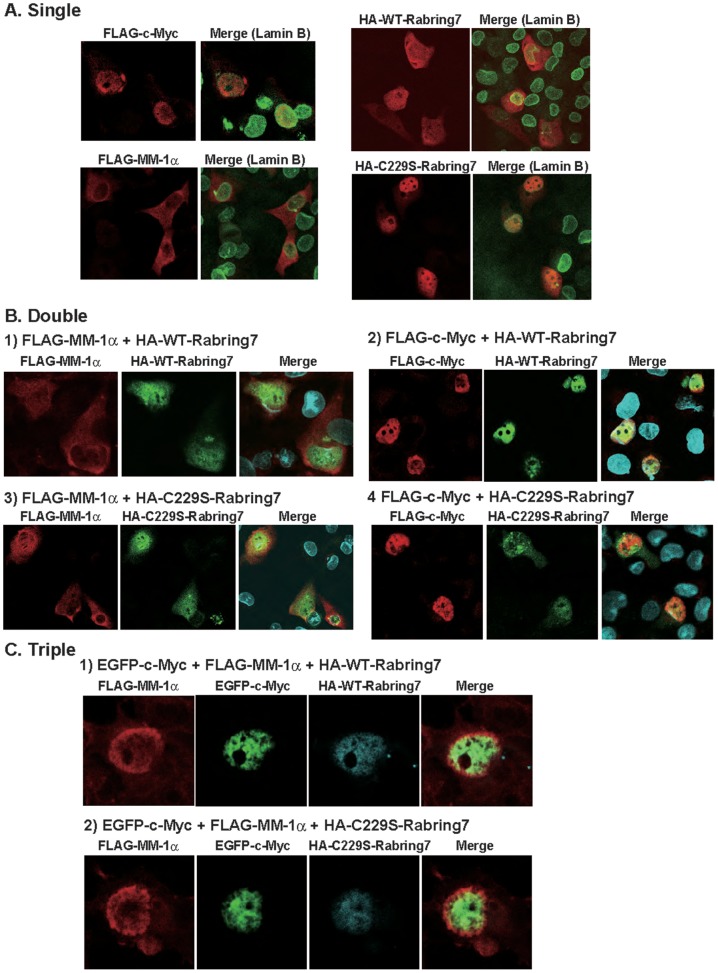
Localization of Rabring7 and MM-1α. **A–C**. H1299 cells were transfected with various combinations of expression vectors for FLAG-MM-1α, HA-wild-type Rabring7, HA-C229S Rabring7 and FLAG- or EGFP-c-Myc by Lipofectamine 2000. Forty-eight hrs after transfection, the cells were fixed with 4% paraformaldehyde and reacted with anti-FLAG, anti-HA and lamin B antibodies. The cells were then reacted with FITC-conjugated anti-rabbit and rhodamine-conjugated anti-mouse antibodies. A: transfection with FLAG-MM-1α, with FLAG-c-Myc or with HA-wild-type Rabring7 or HA-C229S Rabring7 alone. B: co-transfection with FLAG-MM-1α or FLAG-c-Myc and HA-Rabring7 or HA-C229S Rabring7. C: co-transfection with FLAG-MM-1α, A-wild-type Rabring7 or HA-C229S Rabring7 and EGFP-c-Myc.

Since Rabring7 has been shown to be localized in the cytoplasm [24], we then examined the localization of Rabring7 under the condition of the presence of c-Myc and MM-1α. To examine the effect of ubiquitin ligase activity of Rabring7 on localization of c-Myc and MM-1α, C229S Rabring7, a ubiquitin ligase-negative mutant of Rabring7 in which cysteine at amino acid number 229 was substituted to serine [26], was also used. When H1299 cells were transfected with FLAG-c-Myc, FLAG-MM-1α, HA-wild-type Rabring7 or HA-C229S Rabring7, HA-wild-type Rabring7 was localized both in the cytoplasm and nucleus, and FLAG-MM-1α was mainly localized in the cytoplasm. FLAG-c-Myc and HA-C229S Rabring7 were localized in the nucleus (Figure 5A). When H1299 cells were co-transfected with FLAG-MM-1α and HA-wild-type Rabring7 or HA-C229S Rabring7, a part of FLAG-MM-1α was translocated to the nucleus in which both proteins were co-localized, but HA-C229S Rabring7 remained in the nucleus (Figures 5B-1 and 5B-3). When H1299 cells were co-transfected with FLAG-c-Myc and HA-wild-type Rabring7 or HA-C229S Rabring7, both proteins were located in the nucleus (Figures 5B-2 and 5B-4). When FLAG-MM-1α, HA-wild-type or -C229S Rabring7 and EGFP-c-Myc were co-transfected into H1299 cells, however, almost all of the FLAG-MM-1α and HA-wild-type Rabring7 were translocated to the nucleus and the three proteins were co-localized in the nucleus (Figure 5C).

### Effect of Rabring7 on ubiquitination of c-Myc

**Figure 6:**
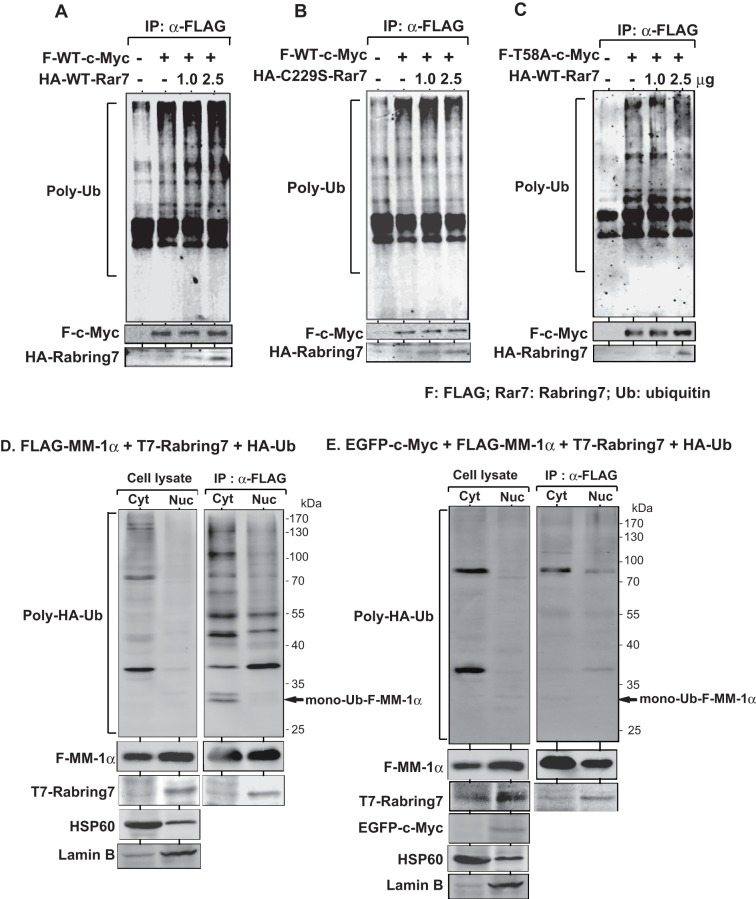
Stimulation of poly-ubiquitination of c-Myc by Rabring7. **A** and **B**. HEK293T cells were co-transfected with FLAG-wild-type-c-Myc together with two doses of HA-wild-type Rabring7 (A) or HA-C229S-Rabring7 (B). Forty-four hrs after transfection, 25 μM MG132 was added to the culture medium, and the cells were cultured for an additional 4 hrs. Proteins in cells were then immunoprecipitated with an anti-FLAG antibody, and the precipitates were analyzed by Western blotting with an anti-multi-ubiquitin antibody. **C**. HEK293T cells were co-transfected with FLAG-T58A-c-Myc together with two doses of HA-wild-type Rabring7 and subjected to ubiquitination assays as described in the legends of Figures 6A and 6B. D. HEK293T cells were co-transfected with FLAG-MM-1α, T7-Rabring7 and HA-ubiquitin. Forty-eight hrs after transfection, total cell lysates were prepared and the cytoplasm and nucleus were fractionated as described in Experimental procedures. Proteins extracted from them were immunoprecipitated with an anti-FLAG antibody and analyzed by Western blotting with anti-HA, anti-FLAG and anti-T7 antibodies. Fractions of the cytoplasm and nucleus were also blotted with anti-HSP60 (SC-6216, Santa Cruz biotechnology, Santa Cruz, CA) and anti-Lamin B (SC-13115, Santa Cruz biotechnology) antibodies. E. HEK293T cells were co-transfected with EGFP-c-Myc, FLAG-MM-1α, T7-Rabring7 and HA-ubiquitin. Proteins were analyzed as described in the legend of Figure 6D.

Since Rabring7 binds to c-Myc and is ubiquitin ligase, the effect of Rabring7 on ubiquitination of c-Myc was examined. HEK293T cells were co-transfected with FLAG-c-Myc together with two doses of HA-wild-type Rabring7 or HA-C229S-Rabring7. Forty-four hrs after transfection, 25 μM MG132 was added to the culture medium and the cells were cultured for an additional 4 hrs. Proteins in cells were then immunoprecipitated with an anti-FLAG antibody, and the precipitates were analyzed by Western blotting with an anti-multi-ubiquitin antibody. The results showed that while the level of ubiquitinated FLAG-c-Myc was increased with increasing doses of HA-wild-type Rabring7, the level of ubiquitinated c-Myc was not changed by C229S-Rabring7 (Figures 6A and 6B, respectively). Since c-Myc is ubiquitinated by Fbw7 in a T58-dependent manner [10], the possibility that T58 of c-Myc also affects Rabring7-induced ubiquitination of c-Myc was examined. As shown in Figure 6C, an increased level of ubiquitinated T58A c-Myc by wild-type Rabring7 was not observed. To rule out the possibility that no effect of Rabring7 on ubiquitination of T58A c-Myc is non-binding of Rabring7 to T58A c-Myc, H1299 cells were transfected with FLAG-wild-type c-Myc or FLAG-T58A c-Myc together with HA-Rabring7. At 48 hrs after transfection, proteins prepared from transfected cells were immunoprecipitated with an anti-FLAG antibody or with non-specific IgG, and precipitates were analyzed by Western blotting with anti-HA and anti-FLAG antibodies. Co-immunoprecipitation experiments clearly indicate that FLAG-T58A c-Myc was associated with HA-Rabring7 at the level similar to that of FLAG-wild-type c-Myc (Figure S1). These results suggest that Rabring7 ubiquitinates c-Myc in a T58- dependent manner.

To examine where mono-ubiquitination of MM-1 by Rabring7 occurs in cells, H1299 cells were co-transfected with FLAG-MM-1α, T7-Rabring7 and HA-ubiquitin. At 48 hrs after transfection, the cytoplasm and nucleus were fractionated and their protein extracts were immunoprecipitated with an anti-FLAG antibody. The precipitates were then analyzed by Western blotting with anti-HA, anti-FLAG and anti-T7 antibodies. HSP60 and lamine B were also stained as a marker for the cytoplasm and nucleus, respectively, and their results indicate proper, but not complete, fractionation between the cytoplasm and nucleus (Figure 6D). The co-immunoprecipitation results showed that T7-Rabting7 was associated with FLAG-MM-1α preferentially in the nucleus and partly in the cytoplasm and that mono-ubiquitination of FLAG-MM-1α occurred only in the cytoplasm (Figure 6D). Furthermore, when H1299 cells were co-transfected with EFGP-c-Myc, T7-Rabting7 and FLAG-MM-1α, mono-ubiquitinated FLAG-MM-1α disappeared along with most of the poly-ubiquitinated MM-1α(Figure 6D). These results along with localization results shown in Figure 5 suggest that MM-1α mono-ubiquitinated in the cytoplasm is translocated into the nucleus and stabilized there to make a ternary complex with Rabring7 and c-Myc, leading to Rabring7-induced degradation of c-Myc.

### Effect of Rabring7 on stability of c-Myc

**Figure 7:**
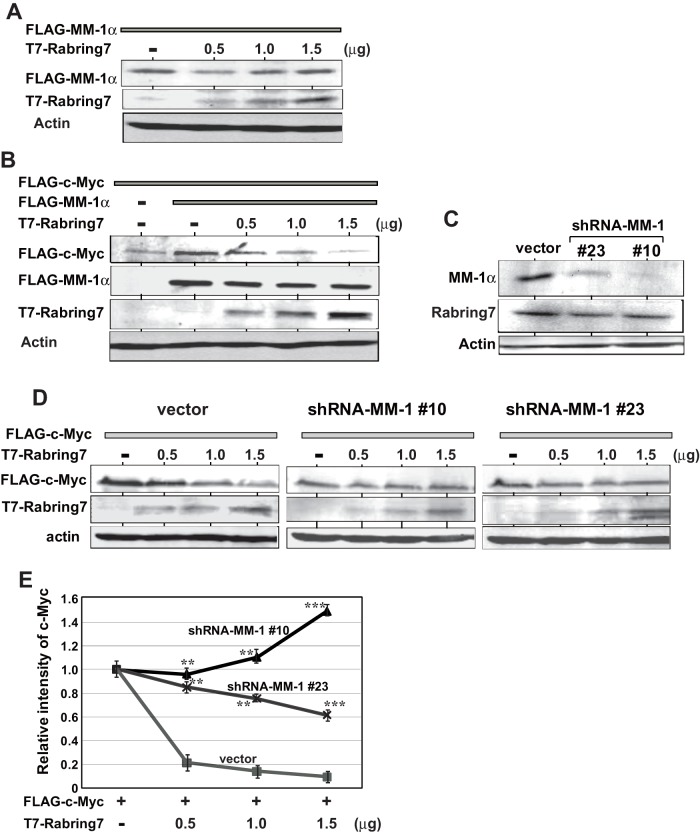
Effect of Rabring7 on stability of c-Myc. **A**. HEK293T cells were co-transfected with a constant amount of FLAG-MM-1α and with various doses of T7-Rabring7, and proteins in cells were analyzed by Western blotting with anti-T7, anti-FLAG and anti-actin antibodies. **B**. HEK293T cells were co-transfected with constant amounts of FLAG-MM-1α and FLAG-c-Myc together with various doses of T7-Rabring7, and proteins in cells were analyzed by Western blotting with anti-T7, anti-FLAG and anti-actin antibodies. **C**. Expression levels of MM-1α, Rabring7 and actin in vector- and MM-1-expressing H1299 cells were analyzed by Western blotting with anti-MM-1, anti-Rabring7 and anti-actin antibodies. **D**. Vector- and MM-1-expressing H1299 cells were transfected with a constant amount of FLAG-c-Myc and with various amounts of T7-Rabring7. At 48 hrs after transfection, protein in the cells were analyzed by Western blotting with anti-T7, anti-FLAG and anti-actin antibodies. **E**. The intensity of bands corresponding to FLAG-c-Myc and actin in Fig. 6D was quantified, and relative intensity of FLAG-c-Myc to that of actin is shown. Values are means ± S.D. n = 3 experiments. Significance: **p<0.01 and ***p<0.001.

Since MM-1α was ubiquitinated, at least mono-ubiquitinated, by Rabring7, the effect of Rabring7 on the protein level of MM-1α was examined. HEK293T cells were co-transfected with FLAG-MM-1α and with various doses of T7-Rabring7, and proteins in cells were analyzed by Western blotting with anti-T7 and anti-FLAG antibodies. As shown in Figure 7A, the expression level of MM-1α was not changed or was slightly increased with increase in the dose of T7-Rabring7. When FLAG-MM-1α was co-transfected with a constant amount of FLAG-c-Myc and with various doses of T7-Rabring7, on the other hand, the level of FLAG-c-Myc was decreased in a dose-dependent manner and the level of FLAG-MM-1α was slightly decreased or not changed (Figure 7B). To examine the effect of MM-1α on Rabring7-induced degradation of c-Myc, MM-1α-knockdown H1299 cells, #10 and #23, and H1299 cells harboring a vector (vector cells) were co-transfected with FLAG-c-Myc and T7-Rabring7, and the expression level of c-Myc was examined. MM-1α expression was decreased by about 60% and 80% in MM-1-knockdown cells #10 and #23, respectively, compared to the expression level in parental H1299 cells, and the expression level of Rabring7 was not significantly changed (Figure 7C). While c-Myc was degraded by introduction of T7-Rabring7 in vector cells in a dose-dependent manner, c-Myc was stabilized in MM-1α-knockdown cells and the level of stabilization of c-Myc was parallel with the level of knocked-down MM-1α expression (Figure 7D and its quantified data (Figure 7E). These results suggest that Rabring7 stimulates c-Myc degradation only in the presence of MM-1α.

### Effect of Rabring7 on cell growth

**Figure 8:**
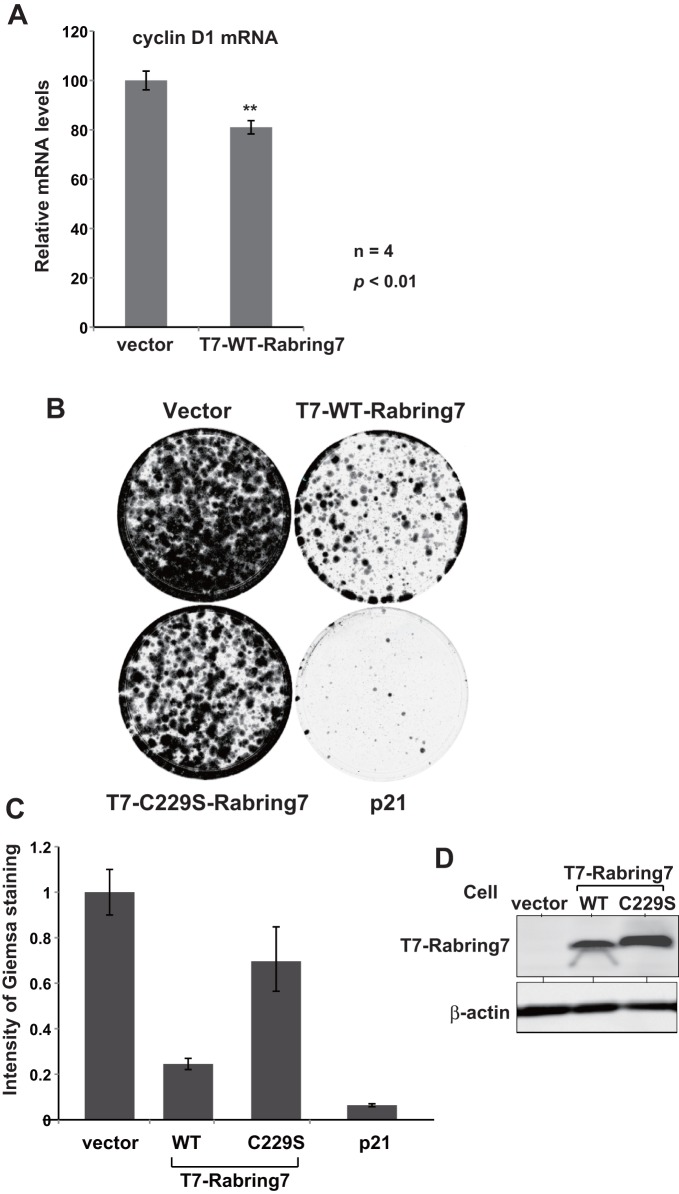
Effect of Rabring7 on cell growth. A. H1299 cells were transfected with an expression vector for T7-Rabring7 or with an empty vector. Forty-eight hrs after transfection, total RNAs were extracted and expression levels of cyclin D1 and actin mRNA were examined by real-time PCR as described in Experimental procedures. Relative expression level of cyclin D1 versus actin mRNA is shown. B. H1299 cells were transfected with expression vectors for T7-wild-type Rabring7, T7-C229S Rabring7 or p21, or with an empty vector. Forty-eight hrs after transfection, cells were added with G418 and cultured for 18 days. Cells were then stained with Giemsa. C. Intensity of Giemsa staining of cells were quantified. D. Proteins were extracted from transfected H1299 cells as described in the legend of Figure 8B were analyzed by Western blotting with anti-T7 and anti-actin antibodies.

To examine the effect of Rabring7 on function of c-Myc, H1299 cells were transfected with an expression vector for T7-Rabring7 or with an empty vector. At 48 hrs after transfection, the expression level of cyclin D1 mRNA, a transcriptional target gene of c-Myc, was examined by real-time PCR (Fig. 8A). The results showed that the expression level of cyclin D1 mRNA was significantly decreased in T7-Rabring7-transfected cells. Since c-Myc promotes cell growth, the effect of Rabring7 on cell growth was examined by colony forming assays. To do so, H1299 cells were transfected with pcDNA3-T7-wild-type-Rabring7, pcDNA3-T7-C229S-Rabring7, pcDNA3-p21 or pcDNA3, and cultured in the presence of G418. p21, a CDK2 inhibitor, was used as a negative control. At 18 days after G418 selection, colonies obtained were stained with Giemsa solution and their intensity was measured. Expression of T7-wild-type Rabring7 and T7- C229S Rabring7 in transfected cells were confirmed by Western blotting (Fig. 8D). As shown in Figs. 8B and 8C, empty vector-transfected cells produced numerous colonies and little colonies were observed in p21-transfected cells. While a number of colonies was extensively decreased in wild-type Rabring7-transfected cells, transfection of C229S-Rabring7 into cells did not obvious effect on colony number. These results suggest that Rabring7 antagonizes functions of c-Myc.

## Discussion

In this study, we first identified Rabring7/BCA2 as an MM-1-binding protein and later found that Rabring7 also binds to c-Myc. Rabring7 mono-ubiquitinated MM-1 without degradation of MM-1 and stimulated poly-ubiquitination of c-Myc. Knockdown of MM-1 expression stabilized c-Myc even when Rabring7 was present. These results suggest that Rabring7 induces c-Myc degradation through mono-ubiquitination of MM-1. The regulation of c-Myc stability by the combination of Rabring7 and MM-1 is a novel mechanism.

MM-1 is a c-Myc-binding protein and represses transcription and transforming activity of c-Myc [11]–[13]. MM-1 also stimulates degradation of c-Myc by binding to the proteasome [22] and plays a role in chaperone function as a subunit of prefoldin [18], [19]. In addition to the MM-1-binding proteins described above, Rabring7 was identified as a new MM-1-binding protein by using co-immunoprecipitation followed by TOF-MS analysis (Figure 1A). Rabring7 directly binds to MM-1 and makes a complex with MM-1 in cultured cells (Figures 1B, 1C, 1D). Although Rabring7 ubiquitinated MM-1 as an E3 ubiquitin ligase, mono-ubiquitination of MM-1 occurred in the cytoplasm more strongly than did poly-ubiquitination of MM-1 (Figures 2C and 6D). Rabring7 is poly-ubiquitinated by Rabring7 itself, and only an EGF receptor is known as a protein degraded by Rabring7 [26]. Poly-ubiquitination of the EGF receptor by Rabring7 has, however, not be shown yet.

Unexpectedly, we found that Rabring7 more strongly binds to c-Myc than to MM-1 (Figure 4A) and that Rabring7 stimulates poly-ubiquitination of c-Myc in a T58-dependent manner (Figure 6). The reason for the weak stimulation of poly-ubiquitination of c-Myc by Rabring7 is that endogenous ubiquitin present in cells, not exogenously added ubiquitin, was used in poly-ubiquitination assays. Rabring7 was localized both in the cytoplasm and nucleus, and MM-1α was mainly localized in the cytoplasm (Figure 5A). It is of interest that a part of MM-1α were translocated to the nucleus in which MM-1 and Rabring7 are co-localized under the condition of a low level of c-Myc expression and that almost all of the MM-1, Rabring7 and c-Myc were co-localized in the nucleus when c-Myc expression level was increased (Figures 5B and 5C). This is the first report of translocation of Rabring7 from the cytoplasm to the nucleus. Although MM-1 is mono-ubiquitinated by Rabrign7 in the cytoplasm, MM-1 is not degraded (Figures 6C and 7). Furthermore, the ubiquitinated MM-1α level was decreased when c-Myc expression level was increased (Figure 6E). From these results, it is thought that mono-ubiquitination of MM-1α is a sign for MM-1α to translocate to the nucleus. There have been several studies showing that mono-ubiquitination of proteins regulates their activities and characteristics, including translocation of the DCN1-like protein hDCNL1 between the cytoplasm and nucleus [29], stabilization of Bcl-2 by Parkin to inhibit autophagy [30], transcriptional repression activity of histone H2A [31], transcriptional activation activity of p53 [32], Smad 4 [33] and Foxo4 [34], and switch from replicative to translesion DNA polymerases by proliferating cell nuclear antigen (PCNA) [35].

When Rabring7 was overexpressed, the expression level of c-Myc was decreased in cells expressing MM-1α and the expression level of c-Myc was increased in MM-1-knockdown cells (Figure 7), indicating that Rabring7 is effective for c-Myc degradation only in the presence of MM-1α, possibly mono-ubiquitinated MM-1. Furthermore, poly-ubiquitination of a T58 substitution mutant of c-Myc was not affected by Rabring7 (Fig. 6C), suggesting that, like Fbw7-induced c-Myc degradation [10], phosphorylation of T58 is necessary for Rabring7-induced c-Myc degradation. Further study is necessary for this issue. Overexpression of Rabring7 decreased the expression level of cyclinD1, a transcriptional target for c-Myc, and decreased cell growth (Figure 8). These results suggest that Rabring7 antagonizes function of c-Myc possibly through degradation of c-Myc.

**Figure 9:**
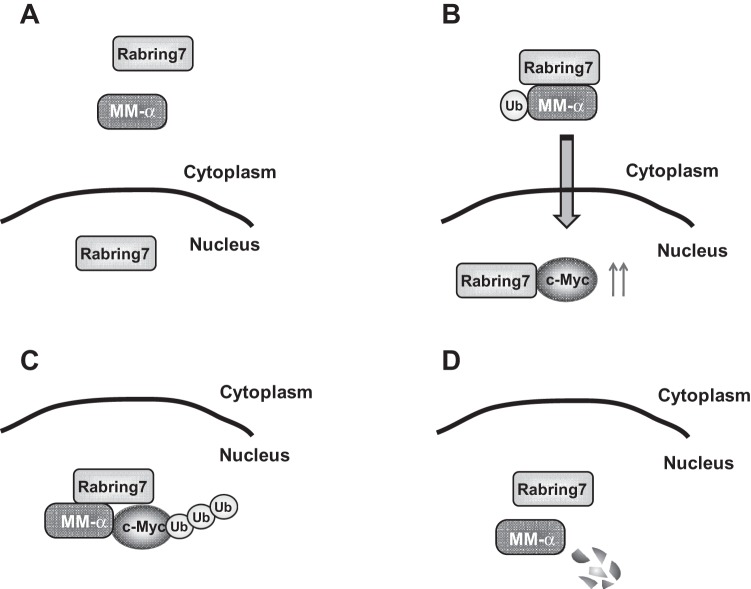
Schematic model of functions of MM-1 and Rabring7 in c-Myc degradation.

Based on the results, we propose the following model of actions of MM-1 and Rabrin7 toward c-Myc (Fig. 9): A. MM-1 is localized mainly in the cytoplasm, and Rabrin7 is localized in the cytoplasm and nucleus in cells under the condition of a low expression level of c-Myc. B. After the expression level of c-Myc is increased, Rabring7 binds to MM-1 to mono-ubiquitinate it, facilitating translocation of MM-1 and Rabrin7 from the cytoplasm to the nucleus. C. Rabrin7 complexed with MM-1 poly-ubiquitinates c-Myc. D. Poly-ubiquitinated c-Myc is degraded by the proteasome system. Since Rabring7/BCA2 is over-expressed in breast cancer cells [25], it would be interesting to examine the characteristics of MM-1 and c-Myc in breast cancer cells.

It is concluded that Rabring7 stimulates c-Myc degradation via mono-ubiquitination of MM-1 and that Rabring7-induced c-Myc degradation is a novel regulation for c-Myc activity.

## Materials and Methods

### Cell culture, transfection and establishment of MM-1-expressing cells

Human H1299, HEK293T and HeLa cells were purchased from American Tissue culture collection (ATCC) and cultured in Dulbecco's modified Eagle's medium supplemented with 10% calf serum. Transfection of plasmid DNAs into cells was carried out by the calcium phosphate precipitation method or by using Lipofectamine 2000 (Invitrogen, Carlsbad, CA) according to manufacturer's protocol. H1299 cells were co-transfected with expression vectors for FLAG-tagged MM-1α and for the hygromycin-resistant gene, and the cells were cultured in the medium in the presence of 400 μg/ml hygromycin for 14 days. Cells that were resistant to the drug were then selected, and expression of FLAG-MM-1α was examined by Western blotting with an anti- FLAG antibody (1∶4000, M2, Sigma, St. Louis, MO). These cells were named F-MM-1-expressing cells.

### Plasmids

pCI-neo-HA-Rabring7 [24] was kindly provided by Dr. T. Sasaki. After digestion of pCI-neo-HA-Rabring7 with *Eco*RI and *Xho*I, the resultant fragment containing Rabring7 was inserted into the respective site of pcDNA3-T7 or pMP-1.

### Western blotting and sub-cellular fractionation

Proteins were extracted from cells in an NET buffer containing 50 mM Tris-HCl (pH 8.0), 150 mM NaCl, 0.5% NP-40, 5 mM EDTA, 0.1 mM *p*-ABSF, 2 μg/ml leupeptin, 5 μg/ml pepstatin A and 1 μg/ml aprotinin or in a RIPA buffer containing 50 mM Tris-HCl (pH 7.4), 150 mM NaCl, 1% NP-40, 0.5% sodium deoxycholate, 0.1% SDS, 1 mM EDTA, 0.1 mM *p*-ABSF, 2 μg/ml leupeptin, 5 μg/ml pepstatin A and 1 μg/ml aprotinin, and the proteins were subjected to Western blot analysis with anti-FLAG (1∶4000, M2, Sigma), anti-HA (1∶1000, Bethyl lab., Montgomery, TX), and anti-actin (1∶4000, Chemicon, Temecula, CA) antibodies. The proteins were then reacted with an IRDye800 (Rockland, Philadelphia, PA)- or Alexa680 (Molecular Probes, Eugene, OR)-conjugated secondary antibody and visualized by using an infrared imaging system (Odyssey, LI-COR, Lincoln, NE). For sub-cellular fractionation, cells were washed twice with cold PBS, resuspended in a buffer A containing 50 mM Tris-HCl (pH 8.0), 150 mM NaCl, 0.4% NP-40, 5 mM EDTA, 0.1 mM p-AEBSF, 2 μg/ml leupeptin, 5 μg/ml pepstatin A and 1 μg/ml aprotinin and incubated on ice for 5 min. The lysates were centrifuged at 1,000×G for 5 min at 4°C and the cytoplasm extract was collected. The pellet was resuspended in a RIPA buffer containing 50 mM Tris-HCl (pH 7.4), 150 mM NaCl, 1% NP-40, 0.5% sodium deoxycholate, 0.1% SDS, 1 mM EDTA, 0.1 mM p-ABSF, 2 mg/ml leupeptin, 5 mg/ml pepstatin A and 1 mg/ml aprotinin and incubated on ice for 10 min. The lysates were centrifuged at 13,000×G for 10 min at 4°C and the nuclear/membrane extract was collected.

### In vitro binding assay

^35^S-labeled Rabring7 was synthesized *in vitro* using the reticulocyte lysate of the TnT-transcription-translation coupled system (Promega, Madison, WI). GST-FLAG-MM-1 was expressed in and prepared from *E. coli,* and GST was cleaved off by digestion of GST-FLAG-MM-1 with precission protease (GE Healthcare Bio-Sciences). Twenty μl of ^35^S-Rabring7 was mixed with 3 μg of FLAG-MM-1 at 4°C for 2.5 hrs in a buffer containing 20 mM Tris-HCl (pH 8.0), 150 mM NaCl, 1 mM EDTA and 0.4% NP40. The mixture was then immunoprecipitated with an agarose-conjugated anti-FLAG antibody (M2, Sigma) or with agarose-conjugated mouse IgG. After washing with the same buffer, the precipitates were separated on a 12% polyacrylamide gel containing SDS and visualized by fluorography. To examine the interaction of Rabring7 with c-Myc, 20 μl each of ^35^S-labeled c-Myc, MM-1 and transthyretin were reacted with 30 μg of MBP-Rabring7 at 4°C for 2 hrs in a buffer containing 20 mM Tris-HCl (pH 7.4), 150 mM NaCl, 5 mM MgCl_2_ and 0.1% NP40, and pull-down experiments were carried out as described previously [11].

### In vivo binding assay

Five μg of pcDNA3-FLAG-MM-1 together with 5 μg of pCI-neo-HA-Rabring7 was transfected into 60% confluent human H1299 or HEK293T cells in a 10-cm dish by the calcium phosphate precipitation technique. Forty-eight hours after transfection, the whole cell extract was prepared by the procedure described in the Western blotting section. Approximately 2 mg of the cell proteins was first immunoprecipitated with an agarose-conjugated anti-FLAG antibody (M2, Sigma) or with agarose-conjugated mouse IgG under the same conditions as those for the *in vitro* binding assay as described above. After washing with the same buffer, the precipitates were separated on a 12% polyacrylamide gel containing SDS, blotted onto a nitrocellulose filter, and reacted with a rabbit anti-HA antibody (Bethyl Lab.) or with the mouse anti-FLAG antibody. Immunoprecipitated proteins were then reacted with an IRDye800- (Rockland) or Alexa680-conjugated secondary antibody (Molecular Probe) and visualized by using an infrared imaging system (Odyssey). To examine endogenous interaction of c-Myc with Rabring7, proteins extracted from HEK293T cells were immunoprecipitated with an agarose-conjugated chicken anti-c-Myc antibody (Abcam, Cambridge, UK) or with an agarose-conjugated chicken IgY (Acris Antibodies, San Diego, CA) under the same conditions as those for the *in vitro* binding assay, and the precipitates were subjected to Western blotting with anti-c-Myc (1∶1000, N262, Santa Cruz, California, CA) and anti-Rabring7 (1∶1000, ab80432, Abcam) antibodies. To examine endogenous interaction of MM-1 with Rabring7, protein extracts were immunoprecipitated with an anti-MM-1 antibody (S-20, Santa Cruz) or with normal goat IgG and subjected to Western blotting with anti-MM-1 (1∶50, S-20, Santa Cruz) and anti-Rabring7 (1∶1000, ab80432, Abcam) antibodies. To examine a ternary complex of c-Myc with MM-1-1α and Rabring7, H1299 cells were transfected with FLAG-c-Myc together with T7-Rabring7 and HA-MM-1 as described above. At 48 hrs after transfection, proteins prepared from transfected cells were first immunoprecipitated with an agarose-conjugated anti-FLAG antibody, and the precipitates were then eluted from agarose by FLAG peptide. About 2 mg of proteins in elutes was again immunoprecipitated with an anti-HA antibody, and the precipitates were analyzed by Western blotting with anti-T7, anti-HA and anti-FLAG antibodies.

### Ubiquitination assay in vivo

HEK293T cells in a 10-cm dish were transfected with 5 μg each of pcDNA3-FLAG-MM-1α and pcDNA3-HA-Ubiquitin by the calcium phosphate precipitation technique. Forty-four hrs after transfection, 25 μM MG132 was added to the culture medium and the cells were cultured for an additional 4 hrs. Proteins were then extracted as described in the Western blotting section and immunoprecipitated with an agarose-conjugated anti-FLAG antibody (M2, Sigma) or with agarose-conjugated mouse IgG. The precipitates were analyzed by Western blotting with an anti-HA antibody as described in the Western blotting section. To examine the effect of Rabring7 on c-Myc ubiquitination, HEK293T cells in a 10-cm dish were transfected with 2.5 μg of pcDNA3-FLAG-c-Myc together with 1.0 or 2.5 μg of pCI-neo-HA-Rabring7 by Lipofectamine 2000, followed by the same procedures as those described above. After immunoprecipitation of proteins with an anti-FLAG antibody, the precipitates were analyzed by Western blotting with an anti-multi-ubiquitin antibody (1/2000, MBL).

### Ubiquitination assay in vitro

Three μg/μl FLAG-MM-1α was incubated in a mixture containing 6 μg/μl ubiquitin, 200 U/ml pyrophospatase, 250 mM Tris-HCl (pH 9.0), 75 mM MgCl_2_, 50 mM ATP, 25 mM DTT and a mixture of E1, E2 and E3 enzymes (Hokudo, Sapporo, Japan) at 37°C for 90 min. Proteins in the mixture were subjected to Western blotting analysis with an anti-FLAG antibody.

### Indirect immunofluorescence

H1299 cells in 6-well plates were transfected with pcDNA3-FLAG-MM-1α together with or without pCI-neo-HA-Rabring7 by Lipofectamine 2000. Forty-eight hrs after transfection, the cells were fixed with a solution containing 4% paraformaldehyde for 210 min at room temperature and reacted with anti-FLAG (1∶200, Sigma) and anti-HA (1∶100, Bethyl lab.) antibodies. The cells were then reacted with FITC-conjugated anti-rabbit and rhodamine-conjugated anti-mouse antibodies and stained with DAPI. Cell images were observed under a confocal microscope (LSM510, Zeiss, Germany).

### Identification of MM-1-binding proteins

Cell extracts were prepared from F-MM-1-expressing H1299 or parental H1299 cells and immunoprecipitated with an agarose-conjugated anti-FLAG antibody (M2, Sigma), and the immunoprecipitates were separated on a 12.5% polyacrylamide gel. After the gel had been silver-stained, bands that were specifically precipitated with the anti-FLAG antibody were cut out, reduced, alkylated with a buffer containing iodoacetamide, and digested with trypsin. The peptide solutions were desalted, mixed with a-cyano-4-hydroxycinnamic acid, and applied onto a target plate. MS/MS spectra were obtained using an Ultraflex (Brucker Daltonics, Billerica, MA, USA) in reflector mode and analyzed with flexanalysis software (Brucker Daltonics). Protein identification was carried out with Mascot software against the database of NIBI.

### Real-time PCR

H1299 cells were transfected with an expression vector for T7-wild-type Rabring7 or with a vector alone using lipofectamine 2000 (Invitrogen). At 48 hrs after expression, total RNAs were extracted from transfected cells using ISOGEN II (NIPPON GENE, Tokyo, Japan), and the expression level of cyclin D1 and actin mRNA was examined by real-time PCR using MiniOpticon system (Bio-Rad). The nucleotide sequences used were following: cyclin D1 sense, 5′-CACACGGACTACAGGGGAGT-3′; cyclin D1 antisense: 5′-CACAGGAGCTGGTGTTCCAT-3′; actin sense: 5′-CCCTAAGGCCAACCGTGAAA-3′ and actin antisense: 5′-ACGACCAGAGGCATACAGGGA-3α′. PCR mixtures were first heated at 95°C for 30 sec, then 39 cycles at 95°C for 10 sec and 58°C for 30 sec.

### Colony formation assay

H1299 cells in 6-cm dish were transfected with 2 μg of an expression vector for T7-wild-type Rabring7, T7-C229S-Rabring7 or p21 or with a vector alone. Two days after transfection, the cells were trypsinized, divided into four 10-cm dishes containing 400 μg/ml G-418 sulfate (Wako Pure Chemical Industries, Osaka, Japan) and cultured for 18 days. Fresh media were supplied every three days. Cells were then stained with Giemsàs solution (Merck, Germany) and intensity of staining was measured.

### Statistical analysis

Statistical analyses of the results were carried out using one-way or repeated measures analysis of variance (ANOVA).

## Supporting Information

Figure S1 **Association of Rabring7 with wild-type and T58A c-Myc.** H1299 cells were transfected with expression vectors for HA-Rabring7 and FLAG-wild-type c-Myc or FLAG-T58A c-Myc. At 48 hrs after transfection, proteins prepared from cells were immunoprecipitated with an anti-FLAG antibody, and the precipitates were analyzed by Western blotting with anti-HA and anti-FLAG antibodies.
(PDF)
