# Supplementary figures and images for: Rabring7 Degrades c-Myc through Complex Formation with MM-1
Source: PLoS One. 2012 Jul 23;7(7):e41891. doi: 10.1371/journal.pone.0041891 (PMC3402419; doi:10.1371/journal.pone.0041891)

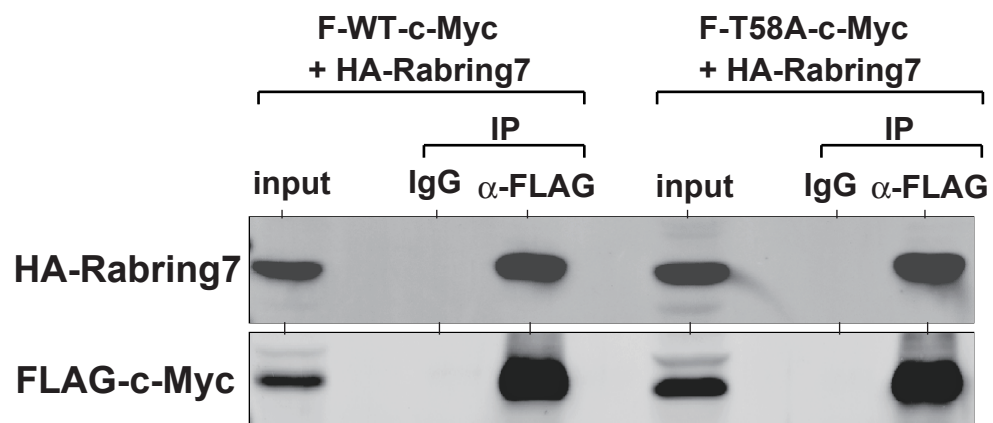

**Fig. S1**

Supplement: Figure S1 — Association of Rabring7 with wild-type and T58A c-Myc. H1299 cells were transfected with expression vectors for HA-Rabring7 and FLAG-wild-type c-Myc or FLAG-T58A c-Myc. At 48 hrs after transfection, proteins prepared from cells were immunoprecipitated with an anti-FLAG antibody, and the precipitates were analyzed by Western blotting with anti-HA and anti-FLAG antibodies. (PDF) [file pone.0041891.s001.pdf]
